# A moving contact line as a rheometer for nanometric interfacial layers

**DOI:** 10.1038/ncomms12545

**Published:** 2016-08-26

**Authors:** Romain Lhermerout, Hugo Perrin, Etienne Rolley, Bruno Andreotti, Kristina Davitt

**Affiliations:** 1 Laboratoire de Physique Statistique de l'Ecole Normale Supérieure, UPMC Univ. Paris 6, Univ. Paris-Diderot, CNRS, 24 rue Lhomond, 75005 Paris, France; 2 Physique et Mécanique des Milieux Hétérogènes, UMR 7636 ESPCI-CNRS, Univ. Paris-Diderot, 10 rue Vauquelin, 75005 Paris, France

## Abstract

How a liquid drop sits or moves depends on the physical and mechanical properties of the underlying substrate. This can be seen in the hysteresis of the contact angle made by a drop on a solid, which is known to originate from surface heterogeneities, and in the slowing of droplet motion on deformable solids. Here, we show how a moving contact line can be used to characterize a molecularly thin polymer layer on a solid. We find that the hysteresis depends on the polymerization index and can be optimized to be vanishingly small (<0.07°). The mechanical properties are quantitatively deduced from the microscopic contact angle, which is proportional to the speed of the contact line and the Rouse relaxation time divided by the layer thickness, in agreement with theory. Our work opens the prospect of measuring the properties of functionalized interfaces in microfluidic and biomedical applications that are otherwise inaccessible.

## Abstract (web summary)

The characterisation of interfacial layers, whose rheology can differ from the bulk, is important for the design of new materials. Here, Lhermerout *et al*. use the dynamics of a moving contact line to quantify the mechanical properties of a polymer thin film.

## Graphical abstract

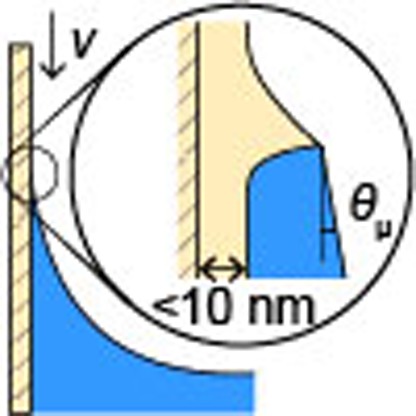

The movement of a liquid is described by hydrodynamics; however, in many cases it also depends strongly on the properties of the underlying solid. For example, it has long been understood that small-scale roughness and chemical heterogeneity on the surface are responsible for contact angle hysteresis as they create energy barriers to motion that pin the three-phase contact line^1^,^2^. For this reason, hysteresis is sometimes used as a measure of smoothness or ‘perfection' of a surface. The difficulty in eliminating all imperfections is illustrated by recent experiments that show how a single nanometric-sized defect produces a hysteresis^3^. The contact line can also move by thermal hopping over these energy barriers, but it has so far proven difficult to correlate the measured dynamics to the surface structure^4^,^5^. In the case of soft solids, the link between the motion of a drop and the deformability of the substrate is better known^6^,^7^. On bulk elastomers or gels, recent experiments have measured the detailed shape of the micrometric deformation induced in the substrate^8^ and quantitatively demonstrated the link between droplet motion and a known substrate rheology^9^. Here, we use the idea that contact line motion contains information about the substrate to now extract properties of an interfacial layer that are not easily accessible to standard rheometers, both because of the very short timescales involved but also because the rheology of an interfacial layer differs from that of the bulk.

Polymers are known to have enormous effects on interfacial phenomena such as slip^10^ and friction^11^ that depend on the microscopic details of the layer. For example, the friction between two solids depends on the molecular organization of the polymers attached to the surfaces^12^. Little is known about how surface-anchored polymers affect the contact angle hysteresis and dynamics. The contact angle hysteresis on smooth, chemically homogeneous solids has been observed to change with the molecular rearrangement of a surfactant monolayer^13^ suggesting that the phase state of surface-anchored polymers may also have an influence on the hysteresis. So far, wetting dynamics on grafted polymer layers—or brushes—has been examined theoretically^14^ and a slowing analogous to the phenomenon of viscoelastic braking^6^,^7^ seen on bulk deformable substrates has been predicted, but it is yet to be observed experimentally.

Here, we show how contact angle hysteresis and dynamics can be used to probe the properties of surfaces covered with PDMS (polydimethylsiloxane) pseudo-brushes of different lengths and swollen by a good solvent. We show that the hysteresis practically vanishes (<0.07°) for a certain polymerization index *N*. This is indicative of the liquid-like nature of the polymer layer, which allows it to hide defects on the underlying solid. We measure the full range of contact angle dynamics on pseudo-brushes, which are <10 nm thick, and use hydrodynamic theory to compute the contact angle at the microscopic scale, which is found to depend on the velocity. The microscopic dynamics are well fitted by a simple model that is based on the viscoelastic deformation of the polymer layer and that depends only on the polymer relaxation time *τ* and the thickness of the layer. In general, this novel combination of experiment and theory—essentially using a moving contact line as a nano-rheometer—can allow one to measure the extremely fast dynamics (*τ*∼100 ns) of surface-bound polymers, which are not accessible to standard rheometric techniques.

## Results

### Hysteresis

**Figure 1:**
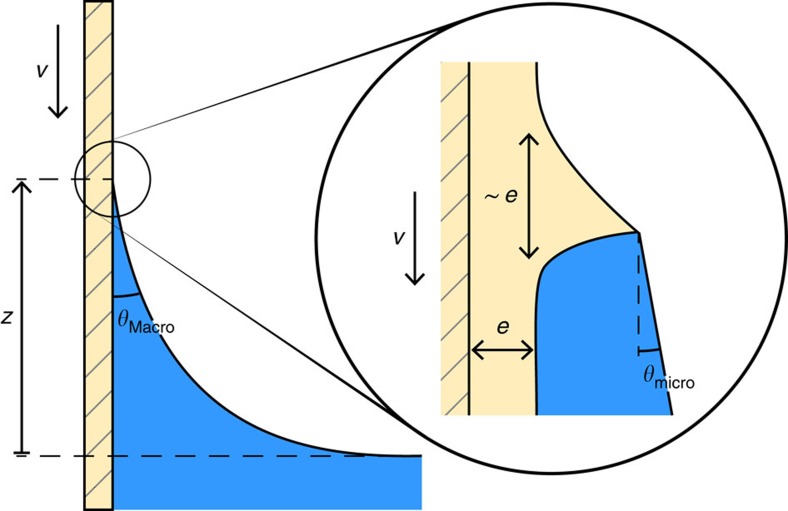
Dip-coating experiment and definitions of the contact angles. In a dip-coating experiment, the meniscus is seen to contact the surface at a so-called apparent or macroscopic contact angle, which depends on the velocity of the contact line, *θ*_Macro_(*v*). Due to the viscous bending of the liquid–vapour interface, this is not the same as the microscopic contact angle *θ*_micro_ in the vicinity of the contact line (zoom). In addition, on a viscoelastic surface, the force exerted by the fluid produces a deformation of the layer immediately below the contact line. The dynamics *θ*_micro_(*v*) reflect the response of the cusp-shaped deformation when the contact line is moving.

**Figure 2:**
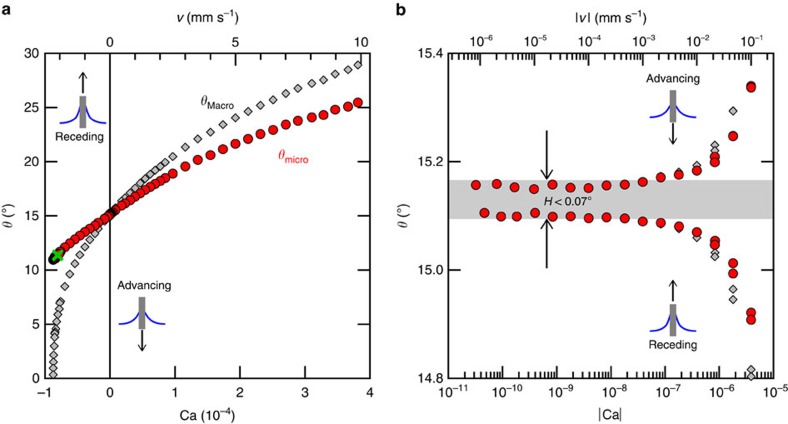
Contact line dynamics on a solid surface covered with surface-anchored PDMS. (*N*=126 with decane). Macroscopic contact angles *θ*_Macro_ (grey diamonds) are determined via optical measurement of the meniscus height, with the solid surface in a dip-coating configuration. A precision of 0.01° in variations of the angle is obtained by averaging over a contact line length of 5 mm. The uncertainty in the absolute angle (global vertical shift of the curves) is 1° and results from the difficulty in accurately determining the reference liquid bath level. Microscopic contact angles *θ*_micro_ (red circles) are calculated from the experimental meniscus height by numerically solving the lubrication equations (Supplementary Fig. 1). (**a**) The dynamics are measured for the full range of contact line speeds, from 1 nm s^−1^ to 1 cm s^−1^, except in the case of a receding line where *θ*_Macro_*=*0 occurs first, here at 2.3 mm s^−1^. The green cross corresponds to the coating transition^29^ (see Supplementary Fig. 1). (**b**) We can detect an extremely small contact angle hysteresis at low velocity (note the logarithmic scale). To within experimental uncertainty, it is ≤0.07° for velocities below 1 μm s^−1^ (shaded region).

Colloquially, contact angle hysteresis is referred to as the difference in contact angle between a situation where the liquid is advancing over the solid and one where it is receding. This definition is imprecise: when the three-phase line is moving, the contact angle depends on the velocity^15^,^16^. The specific velocity-dependence is a signature of the sources of dissipation involved. The two most commonly studied sources are thermally activated hopping of the potential wells created by the molecular discreteness of the solid^17^ or by the chemical or topographical imperfections on the surface^4^,^5^,^18^ and viscous dissipation in the bulk liquid^19^,^20^. The full range of contact-line dynamics are needed to properly characterize the behaviour of a liquid on a surface and to provide a well-defined value for the hysteresis. Here, we measure the dynamics in a classic dip-coating experiment (Fig. 1) where the macroscopic contact angle is determined from the capillary rise *z* according to


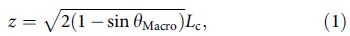


where 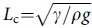, *γ* the surface tension, *ρ* the density and *g* the gravitational acceleration^21^. The surface is plunged into and removed from the liquid bath at speeds *v* between 1 nm s^−1^ and 1 cm s^−1^ to obtain *θ*_Macro_(Ca), where Ca*=vμ*/*γ* is the capillary number and μ the viscosity (Fig. 2). We use decane (*ρ=*730 kg m^−3^, *γ=*23.83 mN m^−1^ and *μ=*0.92 mPa s) as the wetting liquid, which is a good solvent and yields contact angles^22^ of ∼15°.

**Figure 3:**
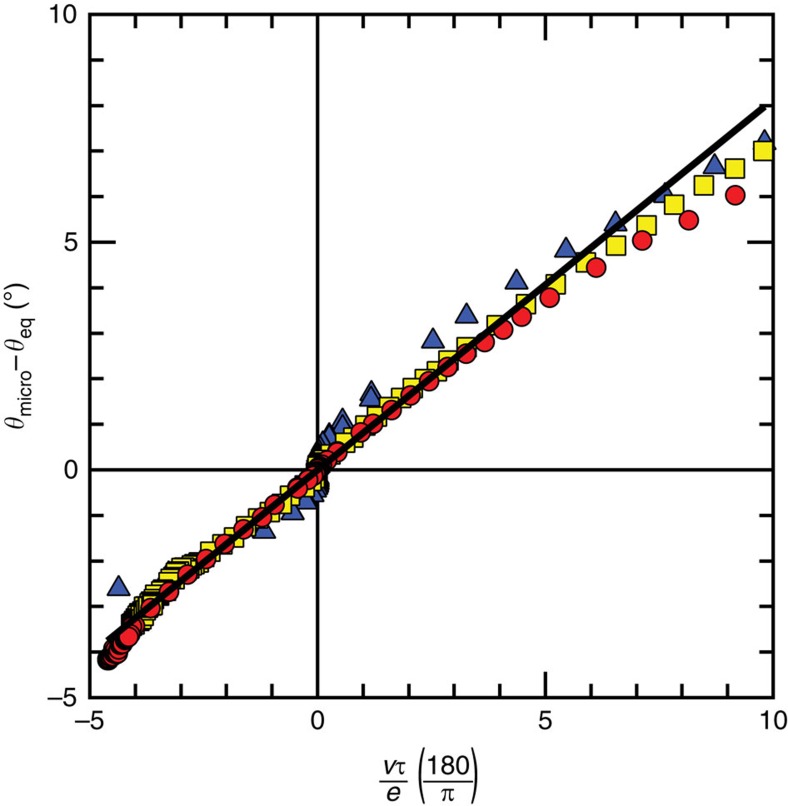
Collapse of the dynamics according to a simple model of viscoelastic dissipation. The microscopic contact angles have been obtained for three different lengths of PDMS (*N=*79, 126, 232 in yellow squares, red circles and blue triangles). By examining the microscopic angle, the bulk hydrodynamic contribution to the dynamics has been removed and what remains is attributable to the viscoelastic response of the pseudo-brush layer under the contact line. In all cases, the hysteresis is small and an equilibrium angle can be identified with little ambiguity as the mid-point between the advancing and receding angles at the lowest velocities; *θ*_eq_=(*θ*_A_−*θ*_R_)/2. Since the equilibrium angle varies slightly with the PDMS length (see Table 1), for comparison between surfaces, deviations from equilibrium are shown. The velocity has been scaled by the thickness of the PDMS layer and the Rouse relaxation time *τ*. The data for different *N* collapse onto a master curve with a best fit (solid line) of slope 0.81.

The hysteresis *H* can be unambiguously defined as the difference between the macroscopic advancing and receding contact angles as *v* approaches zero. We measure the dynamics over seven decades in speed and find that *H*≤0.07° for Ca<4 × 10^−8^ (*v*<1 μm s^−1^) on a PDMS layer of *N=*126 (Fig. 2b). We see that the hysteresis is larger for *N* different from this (Fig. 3, Table 1), but it remains small in absolute terms and compared with surfaces that have been carefully tailored to minimize the hysteresis^23^,^24^,^25^,^26^,^27^. Far beyond entanglement (*N=*1,571) it is 1.60° and at very small *N* (*N=*9) it is 0.97°. For comparison, the hysteresis for water on the underlying silicon substrate without the polymer layer is tens of degrees and it is difficult to measure the dynamics precisely because of the variation across the sample and drift over time. Indeed, this is indicative of the heterogeneity present even on a clean silicon wafer.

It is important to note that PDMS has a low glass transition temperature (*T*_g_*=*−128 °C) and that we work with intermediate polymerization indices (*N=*79, 126 or 232) and a liquid that is a good solvent of the polymer so that the chains are stretched and free. All of these contribute to a liquid-like mobility of the polymer chains that enables it to mask defects on the underlying solid. In this sense, the surface is self-smoothing. Here PDMS has been physically adsorbed to a clean silicon substrate because it gives robust coatings that can be used repeatedly over many months and are very easy to produce. We observe, however, that the adsorption of very short chains (*N=*9) yields a hysteresis that increases with time, which is indicative of the desorption of some polymer and therefore defects in the surface coverage.

### Dynamics of the microscopic angle

In addition to the hysteresis, which gives an indication of the phase state of the layer, the full range of dynamics measured in Fig. 2a can be used to probe the mechanical properties of the polymer chains. For this, we require the contact angle in the immediate vicinity of the contact line, *θ*_micro_. Although *θ*_Macro_ is a useful and well-defined experimental quantity, it is in general not equal to *θ*_micro_ since there is a viscous bending of the liquid–vapour interface when the contact line is in motion^15^,^16^,^19^,^20^,^28^ (see inset in Fig. 1). The distinction between *θ*_Macro_ and *θ*_micro_ is of course not unique to soft substrates, but is a general notion, valid for any solid surface. It is possible to solve the full multi-scale hydrodynamic problem numerically to find the entire interface profile^29^ and relate *θ*_micro_ to *θ*_Macro_. This can be done using hydrodynamic lubrication theory (see Supplementary Methods) and requires only one parameter, namely a cutoff length *l*_c_ determining the scale at which the microscopic angle is defined. This has been extensively used to predict *θ*_Macro_(Ca), assuming that *θ*_micro_ is a constant determined by intermolecular forces. Here, we reverse the method to find *θ*_micro_(Ca) from the measured *z*(Ca) (Fig. 2, Supplementary Fig. 1). When the fluid–substrate interaction is weak and therefore the contact angles are low, hydrodynamic slip-lengths are known to be of molecular-scale^30^. Here, we take *l*_c_ to be the diameter of a decane molecule (0.711 nm). This method allows us to examine the dynamics of *θ*_micro_. In general, this novel approach is appropriate for analysing contact line dynamics provided that additional sources of dissipation beyond that of bulk hydrodynamics are located near the contact line and therefore affect only *θ*_micro_. If hydrodynamics were the only source of dissipation, as one might expect at these velocities and on an ideal solid substrate, then the *θ*_micro_(Ca) shown in Fig. 2a should be constant. Instead, we observe a strong, linear growth of *θ*_micro_ which begins to saturate at the highest velocities.

By examining the microscopic instead of the macroscopic angle, one has removed the effect of viscous dissipation in the liquid. The fact that the microscopic angle depends on velocity and exhibits a dynamics of its own indicates that there is another source of dissipation beyond that of bulk hydrodynamics and whose physical origin is located in the vicinity of the contact line. In fact, as can be seen from the relatively small difference between *θ*_Macro_ and *θ*_micro_ in Fig. 2a, the dynamics is dominated by this effect. Furthermore, we find that the impact on the dynamics grows with increasing *N* (Supplementary Fig. 2), demonstrating unequivocally that it is due to the presence of the polymer layer on the surface and not to inertial effects, for example. For a surface to cause dissipation, it must be deformed. This is seen, for example, when a liquid moves over a bulk viscoelastic material and induces a deformation in the substrate^6^,^7^,^9^. It has also been predicted for surfaces covered with polymer brushes^14^. Following these ideas, below we show how the observed dynamics of the contact angle can be linked to a deformation of the PDMS and consequently to the mechanical properties of this thin layer.

### Deformation of the thin polymer layer

PDMS has a moderate elastic modulus compared with crystalline matter (for the melts used here, it is in the range of 500 kPa) and we expect capillary forces to deform the pseudo-brush layer in the vicinity of the contact line giving a cusp-shaped distortion (inset in Fig. 1), just as on any soft substrate. In contrast to bulk substrates, here the deformation is limited to the scale of the layer thickness *e*, since the latter is much smaller than the elasto-capillary length, hence the characteristic dimensions of the cusp scale as *e*. As a consequence of the liquid-like nature of the layer, qualitatively, one may expect that Neumann conditions determine the angles of the liquid, vapour and cusp. This problem has been addressed theoretically, and it has been shown that Neumann's law indeed applies when one looks at the cusp near the contact line at a scale smaller than *e*, whereas one recovers Young's law when looking at the boundary condition at the meso-scale^8^,^31^,^32^,^33^,^34^.

We consider a contact line moving at constant velocity *v* over such a deformable film. When the contact line moves it drags the cusp along with it. The polymer film is viscoelastic and therefore it cannot be immediately stretched or relaxed to accommodate a cusp that simply translates when the contact line starts moving. Instead, the stretching of chains ahead of the contact line and relaxation of those behind gives rise to a viscous shear stress that tends to rotate the cusp. This is balanced by an elastic stress that limits the rotation. The influence of substrate rheology on contact angle dynamics has only recently been calculated for bulk substrates^9^, here we use scaling arguments to determine the relation between the microscopic contact angle and the mechanical properties of a thin viscoelastic layer. As described above, the experimental microscopic angles have been calculated using lubrication theory assuming a flat substrate, neglecting the presence of the cusp. One can show (Supplementary Fig. 3 and Supplementary Methods) that this is a very good approximation since the cusp size is commensurate with only the smallest length scale of the six decades over which hydrodynamics contributes to the dissipation.

As the contact line and cusp travel at velocity *v*, polymer molecules in the cusp stretch or contract at a strain rate that scales as ∼*v*/*e*. The polymer is subject to a dissipative, viscous stress ∼*ηv*/*e* and an elastic stress ∼*G* Δ*ϕ*, where *η* is the viscosity of the polymer layer, *G* the elastic shear modulus and Δ*ϕ* the rotation of the cusp. The former are related via the relaxation time *τ=η*/*G.* Balancing the local stresses in the cusp yields Δ*ϕ*∼*vτ*/*e*. Near the contact line, the cusp shape is invariant since the Neumann angles are determined uniquely by the interfacial tensions^31^,^32^,^33^,^34^, however it is rotated with respect to the static position^8^,^9^. By geometry, the rotation of the cusp results in a commensurate change in *θ*_micro_ (Supplementary Fig. 4), yielding Δ*θ*_micro_*=*Δ*ϕ*. Alternatively, one can consider the problem at the meso-scale where the film–vapour and film–liquid interfaces are flat and Young's law is valid at vanishing velocity. The driving force per unit length is the unbalanced Young force *γ* (cos *θ*_micro_−cos *θ*_eq_)≈*γ* sin *θ*_micro_ Δ*θ*_micro_, which is balanced by the dissipative, viscous force ∼*ηv*. Recognizing that *γ* sin *θ*_micro_ is the vertical component of the capillary force which is balanced by the elasticity of the layer ∼*Ge*, where *e* is the typical length scale over which the film is deformed, one arrives at the same scaling as above: Δ*θ*_micro_∼*vτ*/*e*. Thus, from the slope of the dynamics in Fig. 2a and the thickness of the layer, one can extract the relaxation time of the surface-tethered polymer chains. For *N=*126 shown in Fig. 2a we find 121 ns.

The mechanical properties of such layers are not well-known. To test the validity of this model and scaling argument, we obtain *τ* from *N* and the monomeric friction deduced from the behaviour of short chains using the Rouse model (see Table 1), and measure *e* by ellipsometry. Specifically, the Rouse relaxation time is calculated from *N* and the monomeric friction coefficient ζ according to 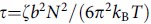, where *b* is the monomer size taken as 0.46 nm, *k*_B_ is the Boltzmann constant and *T* the temperature. A friction coefficient of 9.90 e^−12^ N s m^−1^ is determined from the supplier-tabulated kinematic viscosity of very short-chain melts using 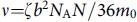, where *N*_A_ is the Avogadro number and *m*_*0*_ is the monomer molecular mass of 74.1 g mol^−1^. The dynamics for adsorbed layers with different *N* then collapse onto a master curve with a slope of order 1 (Fig. 3, and on a log–log scale in Supplementary Fig. 5), indicating that this simple model captures the essential elements required to explain the additional, viscoelastic source of dissipation appearing on surface-anchored-polymer coatings.

From work on gels, it is known that the non-linearity at high velocities (clearly seen in Fig. 2a) comes from the fact that cusp shape is no longer purely elastic as viscous effects begin to grow^9^. At high velocity, these effects dominate. The crossover between the two regimes can be described by a characteristic velocity. Therefore, the collapse of the data for different *N* even beyond the linear regime (Supplementary Fig. 5) shows that the model captures the proper rescaling and not only the correct linear slope.

## Discussion

Here, we show how contact angle hysteresis and dynamics can be used to probe the properties of an interfacial layer. We show that it is possible to obtain a truly negligible contact angle hysteresis by optimizing the choice of surface-tethered polymer and by using good solvents for the polymer. If *N* is too high, the chains are entangled and appear inflexible over relevant timescales. If *N* is too low, the layer is relatively rigid and therefore victim to a hysteresis similar to that on a solid surface. Furthermore, in the case of very short pseudo-brushes there are fewer adsorption sites, which yield layers that are less stable over repeated exposure to a moving contact line. Other methods to attach polymer chains via covalent bonding are well-known and it would be interesting to study the existence of an optimum *N* in this case.

Contact angle hysteresis has adverse effects in any process where one wants to move a fluid over a solid^35^ and its consequences are particularly dramatic when interface areas are large, like in porous media or micro-fluidics. It can translate to a difficulty in moving fluids in applications ranging from oil recovery to imbibition of powders in the food or the cement industries. For this reason, many technical efforts have been expended to reduce hysteresis. To date, record-low values of one degree or less have only be obtained by carefully controlling the self-assembly of monolayers of organic molecules such as silanes and thiols on smooth surfaces^23^,^24^ on small surface areas. By comparison, the simple polymer coatings used here can exhibit a hysteresis of >0.07° and can be deposited on substrates with complex geometries, like microfluidic channels. PDMS can also be adsorbed to glass and metallic surfaces^36^. We anticipate that tailoring the polymer–liquid pair by appropriate choice of polymer, by functionalization of the end-groups, or by imbibition with a good solvent that is immiscible with the partially wetting liquid^37^,^38^, will enable generalizing this method of eliminating contact angle hysteresis.

The same mobility of the polymer chains that yields such an extraordinarily low hysteresis also has major implications for the motion of liquid drops on the surface. Using a combination of experiment and hydrodynamic theory, we extract the dynamics of the microscopic contact angle, and then we use a model to determine the fast relaxation time of surface-bound polymer chains. We validate its use here by rescaling the dynamics for different polymer chain lengths. In general, we propose that a contact line moving on a soft interfacial layer can be used as a nano-rheometer to extract the mechanical properties of a layer that are inaccessible by standard techniques.

## Methods

### Preparing the pseudo-brush

A clean silicon wafer is exposed to oxygen plasma (20 min at a maximum RF power of 30 W, Harrick Plasma PDC-002) then incubated in the undiluted PDMS melt for 24 h at 100 °C: a procedure that produces an irreversibly adsorbed pseudo-brush^39^. Excess, unadsorbed chains are removed by copious rinsing in toluene. The dry thicknesses *e* of the resulting layers are obtained by ellipsometry (average over 3 locations) and do not measurably differ from layers that have been wetted. The thicknesses are between 2.5 and 5 nm and therefore do not change the visual aspect of the solid surface (see Supplementary Video). The PDMS oils in this study were obtained from Gelest, Inc., and used as-received. The polymerization index *N* is determined from the molecular weight MW provided by the manufacturer.

### Data availability

Source data for Supplementary Fig. 1 are provided with the article in the Supplementary Data file. All other relevant data supporting the findings of this study are available from the corresponding authors on request.

## Additional information

**How to cite this article:** Lhermerout, R. *et al*. A moving contact line as a rheometer for nanometric interfacial layers. *Nat. Commun.* 7:12545 doi: 10.1038/ncomms12545 (2016).

## Supplementary Material

Supplementary Information Supplementary Figures 1-5, Supplementary Methods and Supplementary References

Supplementary Data 1 Data from the dip-coating experiment with decane on PDMS N = 126. (The source data for Supplementary Figure 1 which, when analysed as described in the manuscript, yields Figure 2)

Supplementary Movie 1 Comparison of drops sliding on a bare silicon wafer and on a wafer covered with a PDMS pseudo-brush

## Figures and Tables

**Table 1:** Characterization of the PDMS layer.

MW (g mol^−1^)	*N*	*τ* (ns)	*e* (±0.5 nm)	*θ*_eq_ (°)	*H* (°)
5,970	79	57	2.5	15.72	0.17
9,430	126	142	4.0	15.13	≤0.07
17,250	232	476	5.0	12.17	0.32

MW, molecular weight; PDMS, polydimethylsiloxane.

The MW, polymerization index *N* and Rouse relaxation time *τ* for a series of PDMS oils. The thickness *e* of the resulting pseudo-brush layer is measured by ellipsometry. The contact angle hysteresis *H*, defined as the minimum difference between the advancing and receding contact angles, and the equilibrium contact angle *θ*_eq_, estimated as the mid-point between these angles, are found as shown in Fig. 2b.
