# Supplementary material for: A moving contact line as a rheometer for nanometric interfacial layers
Source: Nat Commun. 2016 Aug 26;7:12545. doi: 10.1038/ncomms12545 (PMC5007437; doi:10.1038/ncomms12545)
Supplement: Supplementary Information — Supplementary Figures 1-5, Supplementary Methods and Supplementary References [file ncomms12545-s1.pdf]

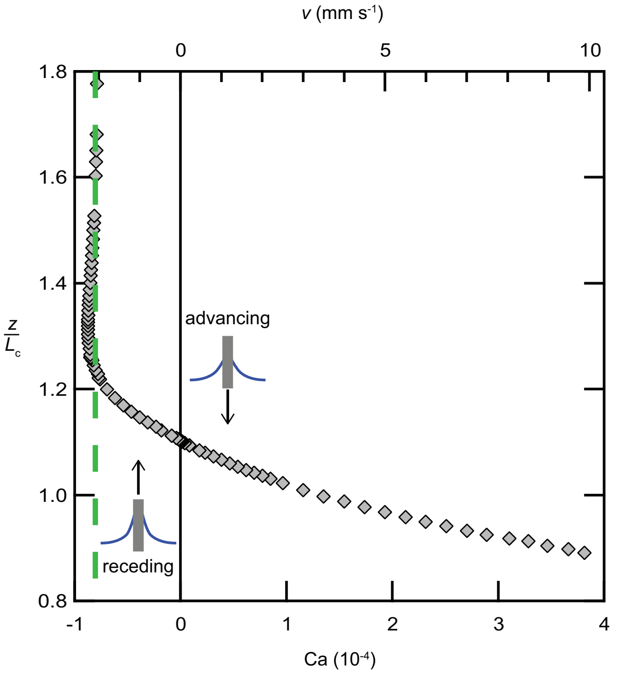

Supplementary Figure 1 | Capillary rise z rescaled by the capillary length L_c as a function of the capillary number Ca. (Corresponds to the experiment in Fig. 2.) Measurements are made in a classic dip-coating experiment with the entire apparatus tilted at an angle of about 10° from the vertical in order to facilitate optical viewing of the contact line, which would normally be obscured by the meniscus on the edges of the liquid-containing cuvette. For an advancing line, and for receding up to the coating transition (dashed vertical green line), the capillary rise z is determined from the stationary meniscus. In practice, to achieve a high precision, variations in z with respect to that at a reference velocity are measured. For the long (10 h) experimental runs required to attain low velocities, it is also necessary to monitor the bulk level of the temperature-controlled liquid bath. At Ca beyond the transition, liquid entrainment occurs and $z\,(\mathrm{Ca})$ can only be obtained from the analysis of transients, i.e., the relaxation of the contact line at the front of the liquid film[1]. Hydrodynamics accounts for the viscous dissipation occurring at all scales and describes the full liquid-vapor interface profile, and therefore also z. In the lubrication theory used here, the flow is non-inertial, Navier slip is allowed at the plate in order to deal with the divergence of the viscous force, and corrections have been included to account for large slopes[2] and the inclined geometry of the plate. The microscopic parameters are the slip length l_c and the contact angle at this scale θ_{micro}. We take l_c to be the size of a decane molecule (0.711 nm) and then numerically solve the problem to find θ_{micro} from each measured z, yielding the curves in Fig. 2.

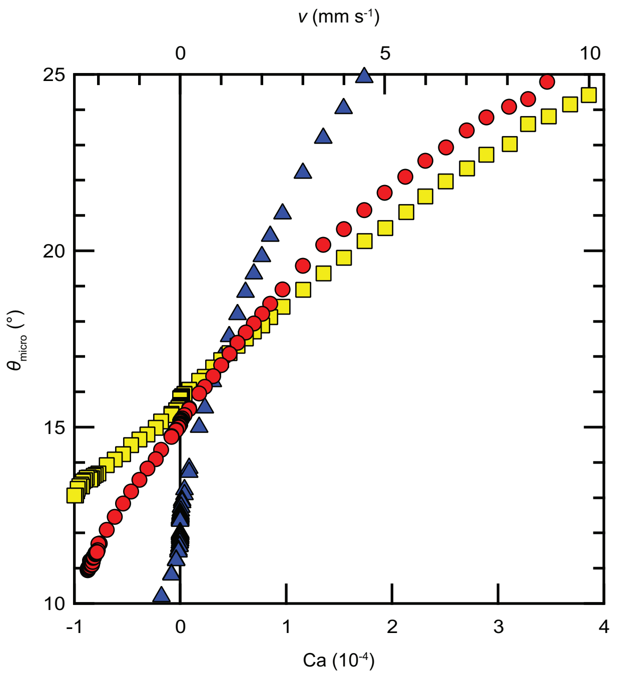

Supplementary Figure 2 | Dynamics of the microscopic contact angle for PDMS coatings with different polymerization indices. (Corresponds to the same data as shown in Fig. 3.) The microscopic contact angles have been obtained for three different lengths of PDMS ($N = 79$, 126, 232 in yellow squares, red circles and blue triangles).

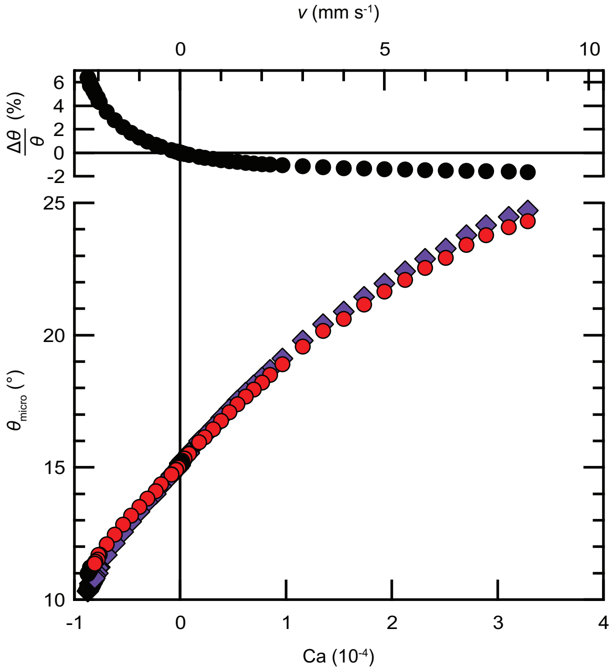

Supplementary Figure 3 | Effect of the substrate deformation on the determination of the microscopic contact angle. (Corresponds to the experiment in Fig. 2.) This figure shows how the microscopic contact angle is affected when one takes the cusp-shaped deformation into account when solving the hydrodynamic problem to extract θ_{micro} from the measured θ_{Macro} (see Supplementary Methods). The modified curve (purple diamonds) is very close to the one obtained for a flat substrate (red circles), with a relative difference of the order of a few percent (black circles). This leads to a commensurately small increase in the slope, validating the approximation of using a flat substrate when extracting θ_{micro}. The fact that the presence of the cusp has only a minor effect can be understood from the fact that it is only a few nanometers in size. Due to the stress singularity at the contact line, viscous dissipation originates from the 6 or more decades of length scale separating the molecular scale from the capillary length, with each decade contributing a comparable amount to the dissipation. The cusp only affects the lowest half-decade and therefore only minimally θ_{micro}.

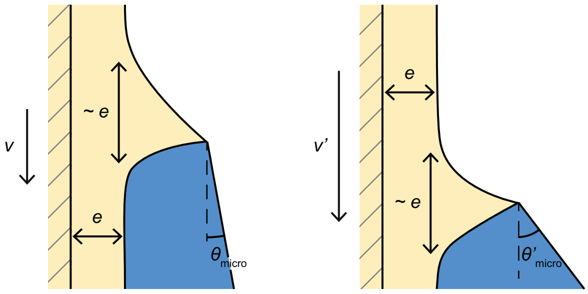

Supplementary Figure 4 | Illustration of a dynamic microscopic contact angle. This figure shows the difference between the cusp-shaped deformation in the polymer film when the contact line is advancing at slower speed v and when it is advancing at faster speed v'. The local shape near the contact line is invariant and determined by Neumann conditions. However, the moving contact line causes a shear stress in the polymer film that tends to rotate the cusp and Neumann triangle. The microscopic contact angle is defined as the angle of the liquid-vapor interface measured with respect to the vertical plate, so a rotation of the cusp and Neumann triangle causes a change in the microscopic contact angle ($\Delta\theta = \theta'_{\text{micro}} - \theta_{\text{micro}}$).

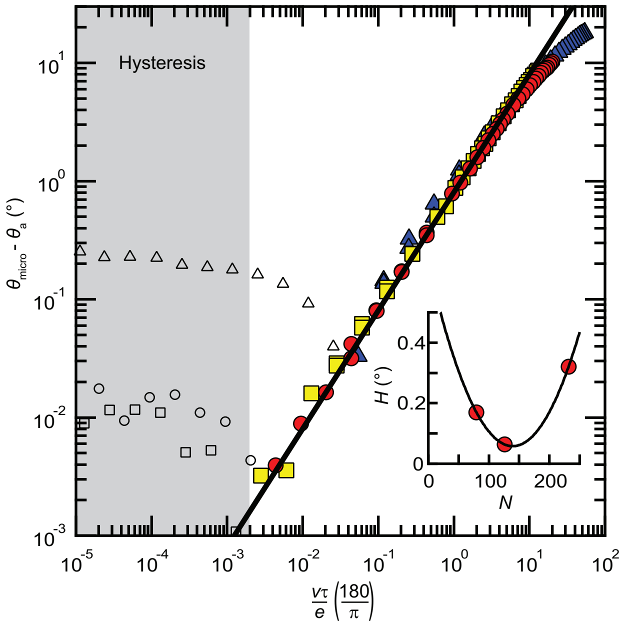

Supplementary Figure 5 | Collapse of the dynamics for different pseudo-brushes shown on a logarithmic scale. (Corresponds to the same data as shown in Fig. 3. $N = 79, 126, 232$ in yellow squares, red circles and blue triangles.) The model accounts for viscoelastic dissipation in the pseudo-brush, but does not describe hysteresis. Here the hysteresis is small, but in order to test the model at extremely low forces — near the origin in Fig. 3 — it must be removed. This has been done by simply subtracting a constant "advancing contact angle" θ_a, determined from the intercept of linear fit to θ_{micro} at higher velocities. A simple subtraction is not entirely sufficient so a "hysteresis zone" still subsists at the lowest forces (open symbols). Nonetheless, where the hysteresis is truly tiny ($N = 79$ and 126) a collapse is seen over nearly 5 decades including at high velocities, beyond the linear regime (black line). Inset: hysteresis as a function of polymerization index.

SUPPLEMENTARY METHODS

Procedure to determine the microscopic contact angle

To determine $\theta_{\text{micro}}\,(\text{Ca})$ from the measurements of the capillary rise $z\,(\text{Ca})$,we solve the lubrication equations extended to arbitrary slopes[2]. We introduce the curvilinear coordinate s along the liquid-vapor interface and write the equations governing the local film thickness $h\,(s)$ and $\theta\,(s)$

$$\frac{dh}{ds} = -\sin\theta \tag{1}$$

and

$$\frac{d^2\theta}{ds^2} = -\frac{\cos\theta}{{L_c}^2} - \frac{2\sin^3\theta\,\text{Ca}}{(\theta - \sin\theta\cos\theta)\,h^2}. \tag{2}$$

The asymptotic solution at the bath ($s \to -\infty$) reads $h \simeq -s$ and $\theta = \pi/2 + c\exp(s) - \frac{\beta}{s^2}$, where the constant c is fixed by the boundary condition at the contact line. For each experimental data point (each Ca), c is entirely determined by the condition that the contact line is located at z; θ_{micro}, the value of θ when $h = l_c$, follows.

Effect of the deformation of the substrate on the hydrodynamics

As described above, the lubrication theory used to find the microscopic contact angle assumes that the underlying solid substrate is flat. To estimate the impact of the cusp-shaped deformation on the determination of θ_{micro}, we numerically solved the modified lubrication equations

$$\frac{dh}{ds} = -\sin\theta, \qquad \frac{dx}{ds} = \cos\theta \tag{3}$$

and

$$\frac{d^2\theta}{ds^2} = -\frac{\cos\theta}{{L_c}^2} - \frac{2\sin^3(\theta - \theta_\text{S})\,\text{Ca}}{[(\theta - \theta_\text{S}) - \sin(\theta - \theta_\text{S})\cos(\theta - \theta_\text{S})]\,(h - h_\text{S})^2}, \tag{4}$$

where we have introduced the coordinate x along the vertical direction, the position h_S and the local angle θ_S of the solid-liquid interface with respect to the flat reference. For numerical computation, the cusp profile is modeled as an exponential function of range e and of slope $\tan\theta_{\text{SL}}$ at the contact line ($x = z$)

$$h_\text{S}\,(x) = e\tan\theta_{\text{SL}}\exp\left(\frac{x-z}{e}\right), \quad \tan\theta_\text{S}\,(x) = -\tan\theta_{\text{SL}}\exp\left(\frac{x-z}{e}\right). \tag{5}$$

For the data shown in Supplementary Figure 3 we have taken $e = 4$ nm and $\tan_{\text{SL}} = 0.6$, values estimated for $N = 126$.

SUPPLEMENTARY REFERENCES

1. Delon, G., Fermigier, M., Snoeijer, J. H., Andreotti, B. Relaxation of a dewetting contact line. Part 2. Experiments. *J. Fluid Mech.* **604,** 55-75 (2008).

2. Snoeijer, J. H., Free-surface flows with large slopes: Beyond lubrication theory. *Phys. Fluids* **18,** 021701 (2006).
